# From Monastic Benevolence to Medical Beneficence: The Inception of Medical Ethics in Wallachia and Moldavia before the Second Half of the 19th Century

**DOI:** 10.3390/ijerph191610229

**Published:** 2022-08-17

**Authors:** Sorin Hostiuc, Oana-Maria Isailă, Octavian Buda, Eduard Drima

**Affiliations:** 1 Department of Legal Medicine and Bioethics, Faculty of Dental Medicine, “Carol Davila” University of Medicine and Pharmacy, 020021 Bucharest, Romania; 2 “Mina Minovici” National Institute of Legal Medicine, 042122 Bucharest, Romania; 3 Department of History of Medicine, Faculty of Medicine, “Carol Davila” University of Medicine and Pharmacy, 020021 Bucharest, Romania; 4 Department of Psychiatry, “Dunărea de Jos” University, 800008 Galați, Romania

**Keywords:** monastic benevolence, beneficence, consent, justice, competence

## Abstract

In Middle Ages, in Moldavia and Wallachia, the healthcare system was almost non-existent, medical practice being the attribute of old women, midwives, charmers, and later monastic personnel. The first elements of medical ethics are identifiable in written texts from the 17th century, associated with a process of laicization of medicine and the appearance of the first combined civil and penal codes (Vasile Lupu’s Law from 1646 and Matei Basarab’s Law from 1652). In the next 150 years, elements of medical ethics were rarely identified, usually in legal regulations, personal letters, or literary works. Starting with the end of the 18th century, associated with the emergence of the position of public physician, detailed regulations regarding the healthcare system associated with an increased number of ethical norms began to emerge. The purpose of this article is to increase awareness to an international audience about the history of Romanian medical deontology and the roots of concepts appertaining to medical ethics in the territories of Moldavia and Wallachia.

## 1. Introduction

Being located at the intersection of three important empires—Russia in the North-East, Hungary/Austro-Hungary in the West, and the Ottoman Empire in the South—the Romanian culture, society, and economics were uniquely shaped, with Western and Eastern, Islamic, and Christian elements. In the Middle Ages, medical practice in Moldavia and Wallachia was mainly performed by old women, charmers, midwives, or other persons without any kind of medical training, who dealt with current and minor medical issues, and monastic personnel. Physicians or even barbers were extremely rare in these countries, and they were, as a general rule, only available to the rules and the wealthiest boyars.

Morality in medicine is a topic as old as medicine itself, as proven by the presence of various ethical-based recommendations in many ancient texts (mostly but not only from the Hippocratic corpus) [1,2,3,4]. Various concepts appertaining to medical ethics were previously gathered under different names—from decorum to deontology or within other disciplines such as law or religion [5,6]. Most Western European and U.S. historians agree that we can talk specifically about medical ethics only after this term was expressly coined by Th. Percival in 1803: “Presentism (n.n. the assumption that because we have a certain concept it must have existed in earlier places or cultures) is anachronistic with respect to medical ethics because earlier medical practitioners who thought about matters we would deem medical ethics thought about them in terms of other concepts—decorum, honor, jurisprudence, prudence—that we no longer take to be directly applicable” [6]. In the last half century, these issues were mainly gathered under the umbrella of bioethics, which is mostly a future-oriented and policy-driven field [6] but is enriched by the use of historical narratives (appertaining to medical ethics, decorum, medical jurisprudence, medical deontology), which may be used to frame the past and expose their attitude to future concepts and ideas [7]. We believe that, in order to reveal the history of a concept either belonging to medical ethics, bioethics, or medicine in general, it is extremely important to analyze its roots and how it developed in a specific, national, and loco-regional context.

Studies regarding the history of medical ethics or the history of concepts and ideas related to medical ethics are scarcely identified in the Romanian literature. Moreover, the history of medicine in South-Eastern Europe is usually less analyzed both in text dealing with Western Europe or Middle East/Ottoman. The purpose of this article is to increase awareness to an international audience about the history of Romanian medical deontology and the roots of concepts appertaining to medical ethics (except for autonomy and consent, which were previously analyzed in depth elsewhere [5]). We will focus our attention on the roots of the concepts of beneficence (monastic benevolence), duty to treat, justice, and the beginning of regularization of medical ethics in Moldavia and Wallachia. We chose not to analyze these issues in Transylvania (the third main constituent of present-day Romania), as it has significant differences caused by its inclusion in the Hungarian Kingdom and later on Austro-Hungarian Empire.

## 2. Materials and Methods

This article is an unsystematized, historical review based on both primary and secondary historical sources. The bibliographical research was performed at the National Library from Bucharest, and the National Institute fo Legal Medicine “Mina Minovici” Library.

## 3. Results

### 3.1. Roots of Medical Ethics before the Second Half of the 18th Century

#### 3.1.1. Monastic Benevolence

Before analyzing the concept of monastic benevolence, we must emphasize that Orthodox Christianity considers that all essential elements needed for understanding morality are available to us since the time of the Apostles: “To make our confession short, we keep unchanged all the ecclesiastical traditions handed down to us, whether in writing or verbally, one of which is the making of pictorial representations, agreeable to the history of the preaching of the Gospel, a tradition useful in many respects, but especially in this, that so the incarnation of the Word of God is shown forth as real and not merely phantastic, for these have mutual indications and without doubt have also mutual significations…Thus we follow Paul, who spake in Christ, and the whole divine Apostolic company and the holy Fathers, holding fast the traditions which we have received” [8]. Therefore, Orthodox Christianity considers implicitly that medical morality has been present since the time of the Fathers of the Church. Technological developments, including medical, do not lead to new moral insights but to new derivations from the well-established ones [6]. Before the 18th century, in Moldavia and Wallachia, the Hippocratic writings, which were fundamental for monastic medical practice at the time, were mostly known to monastic personnel. In the Matei Basarab Law, Hippocrates (Ippocrat) is considered one of the Fathers, his writings being analyzed conjointly with the ones of the Great Athanasie or Alexander, in the section about clerical canons [9]. Hippocratic writings began to be widely known by medical personnel only after the beginning of the 18th century, after the translation of his works in Romanian from Greek [10].

Vasile Lupu’s Law from 1646, published in Jassy, Moldavia, also known as the “Romanian book of learning inspired by imperial and other laws”, is the first secular code of laws having an extended applicability in the judicial practice (civil, canonical, and criminal). It attempted to strengthen the central administration of the country by issuing a unitary legal system, issued in a vernacular language, and covered different domains of legal regulation aimed to challenge the previous, oral, traditional, and the more prone-to-abuse system of the law of the land [11]. The text is mainly based on the works of Prospero Farinacci [12] but also various byzantine laws, including Agrarian Byzantine Law from Leon the Isaur and the Basilicales, and was compiled by a scholar known as Eustratie [13], which also took into account various conceptual changes generated by historical and social conditions of the time [14]. Matei Basarab’s Law (also known as the Revised Law or the Big Pravila) was published in 1652 in Wallachia and represents the first judicial regulations containing a pronounced non-religious part in this country. It is around three times larger than Vasile Lupu’s Law, combining several Byzantine treaties and compilations of canonical and civil law (the Sintagma of Matei Vlastare (1335), clerical book Nomokanon of Manuil Malaxos the Commentary of Alexis Aristen, and the Answers of Anastasis of Antiohia) [13] together with the criminal regulations from the Vasile Lupu’s Law, presented in a different order.

Before the second half of the 17th century, priests and monks were often the only persons capable of providing actual help for the sick in monastery infirmaries. The churches and monasteries of Romanian countries were massive constructions, frequently boasting defense walls, organized with diversified personnel, with ample food supplies to resist long periods during wars, and often had infirmaries as annexes (*bolniță*) [15]. The term *bolniță*, derived from the Slavic word “*bolnicia*”, is defined as a place for caring for the sick. Usually, this was a separate enclosure built at a slight distance from the main monastery, which included a few sanctums, sometimes a small church, and a cemetery. The sanctums were initially destined for the care for the sick monks and were catered by infirmary monks [15]. Shortly after the introduction of infirmaries as annexes to churches and monasteries, they began to provide medical help for others as well. The first reason for this approach was the mandatory duty of monasteries to receive and accommodate whomsoever might come to their doorstep. This imposition is identifiable in numerous church charters, the oldest known in these territories being one found at Bistrița Monastery [15], dated 1494, in which is stated: ”Come all ye who labored and art burdened, for rest I shall grant thee” [16]. Additionally, many churches/monasteries had relics or miracle-working icons. For example, Schitișorul Adormirii Maicii Domnului from Neamț carried such an icon for curing the possessed: “From the village of Crocauca, a man called Tehodor, hath led his five-year-old daughter, labored by the devil and stinting, and when they brought her before the holy icon, shedding tears and begging for avail, before all she hath been cured” [16]. The Bistrița Monastery hosted the relics of Gregory Decapolis, brought here by Barbu Craiovescu, which boasted maximum efficiency for plague epidemics (although it is not specified if this is the only disease cured by it) [17]. Additionally, some monastic institutions gained a reputation for curing various afflictions, attracting people by their fame alone, most likely determined by the current or past existence of monks/priests with somewhat advanced medical knowledge or of religious personalities seen as extremely pious, holy, and inspiring curative abilities through their mere presence. For example, at the church under the patronage of Izvorul Tămăduirii, “many people aspire for sanctimony and qualm redemption” [17].

As a result, infirmaries became health care institutes where clerics as well as the needy and the sick could find relief from suffering. Moreover, village or small-town priests were frequently loaded with medical tasks. Gomoiu says, “Without true healers, the priest was, as I said, not only an everyday counselor—the healer of the soul, but beside the all-knowing ‘crone’, he was also the caretaker in cases of body suffering. Short of special books, even of the kind of the Laws of Matei and Vasile, and having sources forlearning, and believing that all sicknesses are sent by God or the devil, it was also artless, that for their healing, priests resorted more often than not to prayer, to the teachings of the holy apostles and to the seven synods” [18]. Monastic medicine had two main sources of knowledge. The first was represented by medical texts circulating in the Romanian countries, which were, up to the 17th–18th centuries, almost exclusively represented by religious books, containing however certain medical elements mainly inspired by = ancient texts, especially from Hippocratic corpus. For example, the Law of Matei Basarab speaks of the four elements (humors) underlining the Hippocratic theory on human physiology and physiopathology: “the human body is molded from four elements: of the blood, as it were, of the phlegm, of the cough and of the black bile. Thence the blood, for it is warm, is of fire; while the phlegm, for it is cold, is of water; and the cough, for it is wet, is of sky; and the bile, for it is dry, is of earth. And at the time of death, with God’s command, the four elements are shared, and the soul is disjoined” [9]. The second source is represented by popular medicine, Christianized by the association of Orthodox symbols/prayers/percepts. Again, Gomoiu gives us an example in this regard: “among others I remember the way it was written for wildfire (erysipelas nn): he took a hell stone pencil (silver nitrate, nn), circumscribed the burning point, and divided the circle through a cross whose arms went from one brim of the circumference to the other, and in the each one of the four sectors he wrote one of the following - Chr. Na. Vi; then, over the entire swollen region, he applied a thick layer of honey and covered everything with a clean cloth” [18].

Medicine was not a normative attribution of the monastic personnel but rather generated by their condition as leaders of the people from their parishes, which included spiritual, educational, or medical components. The medical intervention was thus generated mostly by the benevolence of the monastic personnel [19], namely the virtue of doing good for people seeking help. This virtue led, in 17th–18th centuries, to the institutionalization of the duty to do good by beginning to be stated in various religious and even laic texts. For example, Antim Ivireanu stated in a book of church teachings from the end of the 17th century, “The priest serving at the Chapel of Holy Healers without silvers, is bound to have an earnest care of the sick from the hospitals, hallowing at the beginning of each moon and baptizing him, and twice per week, on Wednesdays and Fridays, to sing at the Chapel for their mending and redemption and for those terribly sick and for those calling for a prayer” [20]. Matei Basarab’s Law detailed punishments for priests taking blood: “and the priest who shall wean or cut on veins, let him be stripped of priesthood 7, and other teachers say 40 days; and other say to forsake them or to strip them of grace” [9]. The presence of punishments for taking blood suggest that this activity was common in the 17th century; moreover, it points out that some priests, besides medical care and phlebotomy, performed small surgical interventions, such as castration: “any bishop or priest or deacon who shall geld someone by his own hand or by command, or by oneself, he shall be stripped of grace; and if he shall be unhallowed, accursed be he, only if he needs must cut himself to be rid of illness, for such is healing, and not enmity against the body” [9]. These tasks were highly requested in the Romanian principalities, as barbers were very rare at that time; furthermore, among the few identified by our historiography, most of them had an extremely poor skill set, with a series of authors concluding that it was better for the sick to be treated by the peasants with empirical remedies than to be treated by such charlatans [21].

The medical monastic activity remained the fundamental medical institution until the mid-seventeenth century, when it began to be slowly replaced by the one performed by actual physicians. This moment is suggested by the manner in which the term medicine man is taken from Vasile Lupu’s to that of Matei Basarab Law [21]. *Vraci* (medicine man), a term of Slavonic origin was used for the first time in the Coresi’s Scheean Psalter [22], one of the first texts written in vernacular Romanian. Before the second half of the 17th century, the term “*vraci*” had a twofold meaning both an actual medicine man as well as a healer of the soul: “So let us go see the medicine man (*vraci in the original text*), Jesus Christ… for he alone is our reliance and refuge” [22]. Vasile Lupu’s Law, drafted in Iasi in 1646, used the term “vraci” as a synonym for “physician”: “He who shall not call on the medicine man (*vraci*) for his woman’s sickness shall bargain for all kinds of soothing mends” [23] or “When it shall be revealed that the wounded called not upon the medicine man (*vraci*)” [23]. In Matei Basarab’s Law from 1652, which almost entirely copies Vasile Lupu’s, some articles referring to physicians maintain the old terminology, while others use the term doctor: “and again the doctor plies his needle and delivers people from the grasp of death” [9]. In monastic texts, the term “*vraci*” continued to be used, but its connotation remained mostly religious.

During the 17th century and onward, names of physicians, initially foreign and then Romanian, became increasingly frequent, and the medical act became a predominantly laic activity. Along with the laicization of medicine, it started to be regulated on two main levels. Indirectly, there are laws providing a series of attributes for the physician within certain judicial activities. This stage began with the Laws of Vasile Lupu and Matei Basarab, which from the 17th century were also the first texts in which are identifiable proto-elements of medical ethics in the Romanian medical historiography. Besides these, indirect elements, a direct component, represented by legal norms, and codes began to specifically regulate medical, hospital, and social care activity since the first half of the 18th century, and became predominant at the end of the century.

#### 3.1.2. Distinctive Elements for the Duty to Care

The indirect regulation level mentioned above did not bring forward elements to suggest the mandatory aspect of the duty to care for patients by physicians. This duty belonged to other people, who had a general duty to care for the sick on multiple levels, from which the professional component provided by a healthcare specialist was only a small part. For example, in the Vasile Lupu’s and Matei Basarab’s Laws, it is stated that the husband is obliged to care for his wife: “He who shall not call on the medicine man for his woman’s sickness shall bargain for all kinds of soothing mends and all other nourishments in longing, and if she doth die of such sickness, the man shall lose all avails from his wife’s dowries, or if she bestowed anything upon him, that shall be seized as well, or if any other worth that be, as is there customary, that is to say, everything he has” [9]. Interestingly, the case vice versa does not apply: “If the woman shall not call upon the medicine man for her husband’s sickness, or disregard him, she shall not lose her dowry bestowed upon by her man” [9]. Therefore, the primary duty to care resided within the family core, with the healthcare specialist being only an aid. In another article from the same laws, it is stated that the killer is obliged to pay the family of the person killed all expenses generated by the murder, including medical fees: “The killer, save for those punished by death, is yet to pay the dead man’s kinsmen with all tolls paid, that the medicine man was paid and other coin tarnished upon his wounds” [9].

The lack of legal and ethical regulations for medical activity, associated with an extremely poor scientific level of healthcare practice, determined some notable personalities in those days to write stories and reflections to emphasize the need for benevolence. Thus, Nicolae Mavrocordat, ruler of both Wallachia and Moldavia on several occasions at the beginning of the 18th century, wrote, “It behooves that wounds and cuts be treated anon with a gentle hand, employing as poultices most of all for those in suffer so as to cast out any doubt of coarseness” [24]. Antim Ivireanul, in Sermons (*Didahii),* said in turn that “The outright and wise physicians strengthen the praise of their craft, and seek not to mend suffering with steel and fire, as the laws of war go, but only those suffering treated with comforting touch and sweet remedy shall learn the sick man’s cure, and to halt all fearful commands of the healing craft, and some days of healing and nourishing meals, easing pains, to show remedy to the man stricken with sickness” [25]. We see from these texts that the patient’s wellbeing and harm minimization were normative attributes of the medical practice: being encompassed in the concept of benevolence.

The gentle hand (which is loosely equivalent with the current concept of non-maleficence) was not seen as mandatory but seen rather as an alternative to a more violent treatment. Thus, again, Antim Ivireanul said, “This steadiness is forevermore the shepherd’s duty to bestow upon his sheep; and if a sheep is crippled, unwavering of its unwillingness, it should be tied down and the rotten flesh carved out, cast away and remedy be dished out, minding the hurls of its legs wherewith it can encroach upon” [26].

Suffering was considered as inextricably linked to treatment efficiency; both doctors and patients considered suffering to be a normal step toward healing. In a different text, Antim Ivireanul stated, “What toll does one pay to endure winter frost, endure summer heat? In sickness, how much does one spend, how much does he suffer, endure, so as to gain what was lost? He takes no notice in spending his wealth, nor tasting bitter medicines, nor quenching his thirst with queasy drinks, not letting his blood; and there is no thing a man will not do for his health and for his fleshly salvation. From this one might learn how sweet hardiness is, and how loving of himself man is” [27].

Although it is obvious that the physician was called to cure the patient or at least to relieve his suffering, these do not seem to be the explicit purposes of the medical act. Physicians used to treat the sick with their own medicines, oftentimes common for various afflictions; sometimes, they even identified the disease, but the actual healing was deemed to be in the hands of Divinity, as He was the one who sent them: “Sicknesses that come from the people is not alone, but many diseases come from God for the punishment of sins (…) And when someone is stricken with sickness, one shall call for the physician, call upon him to perceive the disease… As the diseases are not remedied nor cured by medicines or healing poultices, but let us concede to God, for it is He who holds the power of hardiness and dying and living” [9].

#### 3.1.3. Justice

Social inequity, characteristic for the Middle Ages, generated a high inequity regarding access to medical services. Common folk only had access, in the best cases, to barbers, charmers, or monastic staff with vague medical knowledge. Moreover, until the late-16th century, numerous situations occurred in which neither the great boyars nor even the voivodes had a permanent physician in court to ensure medical befitting their rank. Evidence of this is provided by numerous correspondences with foreign princes asking for physicians or surgeons. For example, in 1507, Radu Vodă, a Romanian voivode, sent a letter to Kronstadt (Brașov) asking for a master physician skilled in healing foot diseases: “And I appraise Thee as mine noble and earnest brethren, that I heard tithe of a master physician skilled in healing foot sicknesses. Thence I call upon and beseech thee to send this man forth with mine own man Nanciul. And if God wills it, to learn the sickness and bring avail, and he shall not come at a loss, nor will Thyselves” [28,29]. The fees for which physicians were brought to the Romanian principalities were extremely high but directly proportionate with the risks of coming to these realms: “il Signore Principe di Moldavia mi fa nuova et efficace instanza per la mia venuta al suo servitio, offerendomi Reali 1500 per stipendo annuo et 300, di donativo per una volta tantum, per mettermi al ordine…” [29] said the physician Andrea Scogardi in a discussion held with ambassador Contarini of Venice in 1640. The sum was large enough so that in 3–4 years, he could return to Venice without financial worries for the rest of his life. Aside from inequity of access to medical resources, there was also an inequity in treatment. Thus, Nicolae Mavrocordat deemed as normal to treat a higher-social-status patient with greater care and gentleness: “Even the sons of doctors use not the same medicine for common folk as they use for people worthy of noble respect; for they serve the latter with more gentleness and wealthier therapeutic means” [24].

### 3.2. First Attempts to Regularize Medical Practice from the Second Half of the 18th to the Second Half of the 19th Century

At the end of the 18th century, significant elements of public health began to be identified in legal regulations. This shift was generated by an important transfer of knowledge from the Western Europe (mainly Austria but also Prussia, Italian region, or France), where many wealthy persons from the Romanian principalities immigrated and brought back Enlightenment ideas, especially regarding the universality of human rights. Many attended the Viennese School of Medicine, where the service of public medicine was already implemented by Gerard van Swieten, based on the Boerhaave’s clinic model [30,31]. This led to the opening of the first hospitals: Colțea in 1695 [32] and Pantelimon in 1735 [32] in București and St. Spiridon in Iași in 1704 [21] and was associated with a quick recovery of delays in practical medicine compared to Western Europe. For example, smallpox vaccination, presented by Janner in 1798–1800 [21,33,34], was widely introduced in Bucharest in 1800 by the physician Constantin Caracas [21,34].

#### 3.2.1. The Institution of Public Physician and Their Duties toward Patients

One of the most important factors in increased access to healthcare was represented by the institution of public physician. It was initially identifiable along with the administrative organization made by Alexandru Ipsilante in Wallachia and Grigore Ghica in Moldavia during 1777–1778, who founded the Charity Box (and Box of Mercy, respectively), where funds from different resources were gathered with the purpose of aiding the poor [32]. These funds paid for public physicians to treat those without financial resources necessary for settling the medical consult and treatment. The first doctor remunerated from the Charity Box was in Craiova: “when a doctor be charged with a heavy leaden family, he shall be granted further 200 thalers from the Box…” [32]. In Moldavia, a charter from 1778 given by Constantin Moruzi specified the following: “Nevertheless, the chosen physician shall be ready at all times, not only in tending for the prosperous, but also in curing the impoverished, content with whatsoever a man shall award thee, as befitting his capacity and station, and he shall seek the needy costless and be content with his purse” [35]. In 1800, Alexandru Ipsilante, ruler of Wallachia, obliged all hospital physicians from Bucharest to provide free consultations and treatment for the poor and only take money from the wealthy. If a wealthy patient refused to pay the physician, he was still obliged to treat; afterward, the physician could petition the state to recover the debt:

“To you minister of public administration, we hereby appraise you on the physicians we have here, that truly within hospitals lies their duty, but are indebted to look after other common folk for their distress, and if this command is discarded, and we hear of instances where one was stricken by sickness, and called upon a physician, and if the latter shall slack, or be the cause of other mishaps, and are unwilling to go, nor willing to seek the ill stricken, until the need arises; henceforth, we hereby command thee to call upon all such physicians who hold a place, and tell them and show them this as Our great command, and to obey as such, for if we should learn of disobedience, our wrath shall fall upon them. Whosoever shall fall ill whilst a doctor be at hand, arduously shall he go the great and small, in due time, to tend to each and every man of the common folk, as befitted, and until he is restored to steadiness, and from those impoverished, and perceived as such, no coin shall be asked, for this is where his monthly wage is bound, and for it is not befitting to wait for payment from the ones scant of coin or not to seek out his sickness. And from those with wealth… it is befitting of them and they shall pay for the physician’s troubles and thank him as is due, and whence a few be powerless to pay, or see it not fit to settle the physician’s troubles and thank him as is due, the Physician shall not cease his approach and seeking of the sick man, and for such Physician to be warranted and be deemed without fail, he shall come and show thee, and you take it upon thyself to inform us, and that household shall submit to command, and provide whatsoever is befitting” [36].

In the following years, we are provided with a series of contracts between physicians and the public administration, establishing their rights and obligations. Thus, in 1805, the physician Stavri Moscul signed an agreement with the House of Physicians from Moldavia, which states:

1. “I, the undersigned, consent to be the doctor of the town of Iasi and undertake to come to each and all households with the sick in different states, being called upon, without a word (without resistance).
2. At any hour and for any sick man who calls upon myself, I shall come, and in any way strive to relieve the sick man after mine own skill, to restore his steadiness and for this I shall not ask for payment from the sick man, but be content with whatsoever the suffering shall deem worthy of repayment, and for those helpless to repay me, I likewise undertake to seek out their sickness, even spending on behalf of the sick.
3. If such happens that a boyar (lord) falls ill in his village, I undertake to journey there, with the knowledge of the tutors of medicine (vestrymen) and upon my arrival to invest mine entire strength in restoring the sick man’s steadiness. I consent to receive a yearly wage of 4200 lei, at every moon’s turn.
4. If I am to be dissatisfied with mine own service in the town and seek to leave service, thence I shall inform the medical tutors (vestrymen) six moons before, so as they might find a befitting physician in my stead in due time, and if the medical Agency shall be dissatisfied with mine services, they shall notify me six moons beforehand” [37].

In 1817, in a document regulating medical activity in Bucharest hospitals, the attributes of each person involved in public health care were clearly defined. Thus, for instance, surgeons must do the following:

“Art 20. The surgeon shall tend to the wounded and lend all aid abased on his craft, he shall counsel and teach those within the hospital, charged with watching over the sick.”

“Art 21. The surgeon and the chief of the clinic shall acknowledge all sick and shall see that the medicine ordained and needed foodstuff, be given on time, as assigned by the physician” [38].

Aside from the general medical public assistance provided free-of-charge for the poor, in the first half of the XIX century, a series of other medical services were implemented, such as general and free vaccination, dental care, or pharmaceutical services. Thus, regarding general and free vaccination, the physician Constantin Caracas wrote, “Towards the year 1800…. we began the undelayed vaccination, without opposition from the residents, so that in 1802, vaccination was general in the entire town (Bucharest, nn.) as well as in other towns” [39]. In Moldavia, smallpox vaccination was implemented in 1803: “Ale Morusi, Hesse, 1802, introductae reniterentur. Idem Princeps de Moldavia optime meritus Doctorem Frolich vaccinatorem constituit de toto principatu, qui omni, quicunque desideravit, gratuito implantavit, sicque factum est, ut variolae longe minores inducant strages et Moldavi ineptum illud variolarum prophilacticum…” [40]. A document from 1832 details the contract of a physician named Vilhelm Maer to provide free dental care, for which he received remuneration from the Bucharest Physicians Committee: “For tending the poor suffering with their teeth, who come every day with broken teeth and other tooth sores…” [41]. Regarding pharmaceutical care, in a town charter from Bucharest regarding the duties of every physician toward their patients, is stated that pharmacies have a duty “to tend to the poor stricken with sickness without payment for his toils” [42]. In 1830, Kiseleff issued a law according to which a pharmacy in Bucharest would offer free medicines to the poor if they should come with a prescription from a doctor (“from a physician’s counsel”) [43].

The implementation of public medical care, although mandatory in theory, suffered numerous deficiencies in practice. The first problem limiting its implementation was a lack of medical schools in the Romanian principalities before the second half of the 19th century. This caused the number of physicians to be far below the actual needs of the population, as the degree was only obtainable in foreign faculties inaccessible to ordinary people, with the medical practice being left in the hands of pseudo-medical staff (barbers, midwives, pharmacists) who practiced it far below the standards of the time. To supplement the number of medical staff, three fundamental actions were implemented: the construction of schools for medical personnel (initially medium sanitary schools and later on universities), extremely generous subsidies for doctors accepting the job of public physician, and granting scholarships for medical studies abroad. To limit medical services performed by charlatans, the doctors were obliged to hold certificates attesting graduation from a recognized medical institution.

Another major issue that was often discussed in the time was the incorrect use of health funds, which were distributed to a very low number of potential patients. For example, Barbu Știrbei felt the need, in an address to the Ministry of the Interior (Inner Department), to stipulate that “so as not to needlessly squander public moneys…with regards to the construction of a county venereology hospital” [44]. The preconceptions of ordinary people, for which the hospital was a very shameful place to go, was another issue limiting the potential usefulness of the institution of the public physician. This preconception was generated by the fact that the initial lexical meaning of the word hospital in Romanian was “the house of the surely sick” [9] or “the house of the pauper and the sick” [9], a place where society’s scum was brought. This meaning was also identified in Vasile Lupu’s Law: “He who consigns his sickly son to a hospital, shall loose his forbearing. Likewise shall become of the son, who consigns his father to a hospital” [23]. Additionally, the physicians often considered beneath their dignity to visit those from the impoverished beds of society. This fact led many rulers to threaten those failing to perform their service duties. Thus, Alexandru Ipsilanti uttered in 1787, “to call upon all such physicians who hold a place, and tell them and show them this as Our great command, and to obey as such, for if we should learn of disobedience, our wrath shall fall upon them” [36]. In another document from 1815 concerning the lists of doctors and their duties, it is stated that “Physicians and surgeons drawing their wage from the Vestry shall all be bound without demur to see any impoverished sick man herein this settlement, without the smallest reward, obeying the Vestry and bolstering to their duties, and whensoever they shall not run to the impoverished, then their disobedience be revealed to the Lordship, so they may be reprimanded” [17]. In 1813, the ruler Caragea withheld the salary of all Bucharest physicians due to them fleeing the city after a plague outburst: “I hath ordained wages to doctors of this settlement, not for engagement alone, but to show with deeds and not astray from the settlement when called upon for sores and sickness… we command thee, that henceforth when each one of them went amiss from this settlement (bez doctor Constantin Caracas and Doctor Constantin Filipescu), to be destitute of wage” [17].

#### 3.2.2. Duty to Care, Beneficence, and Non-Maleficence

During this stage, the duty to care began to be regarded as a main purpose of the medical care, with the healing of the patient not being left at the mercy of the Divinity. This duty to care, which was distinct from the virtue to do good, can be seen as equivalent to the current concept of beneficence.

There are numerous cases in which doctors were involved in patient poisoning either directly or indirectly. For example, referring to physician Ioan Muralt (Giovanni Moralto), as of 29 March 1600, the commissioners of Emperor Rudolf II uttered, “This godless man should stand trial and punished by crucifixion, for it was he who concocted the poison to be given to Duchess Mary Christine” [45]. As the situation was relatively common, the Laws provided punishment by death for the doctor administering the poison with homicidal purposes: “The physician who bestows the poison upon the son to poison his father, that shall be his undoing, lop off his head” [9]. In Moldavia, a document from 1813 clearly stated the doctor’s duty of helping the sick, which is a duty derived from the principle of humanity (beneficence): “These doctors, surgeons and midwives shall henceforth be indebted, and those foreordained this day and those to be on another day, shall expeditiously tend to the needs and calls of boyar, and common folk and poor and weak alike, towards such the evangelical word indebts them, for it is we who are strong that ought to carry the burden of the weak. Now, therefore, this reckoning, founded on human heartedness, it is for them Vestryman boyars to make known to physicians, surgeons and midwives” [35]. The physicians had the duty to avoid maleficence caused to their patient’s health by either an action or omission: “None dare cause distress, or delay the journey for the morrow, and imperil the lives of men, but with their own carriage or on foot to run to the diseased when such is stricken by sickness and exposed” [35]. Midwives were prohibited from leaving the patient (woman after childbearing) until all potential medical problems were eliminated: “No midwife shall forsake the childbed before she warrants that chance of blood oozing has passed” [46]. As a result, the doctor–patient relationship was not optional for the public physician, as his duty was to answer any call, similar to the duty of emergency room physicians today. If the physician was not available, it was his duty to have a colleague take over his function for the duration of his leave: “If perchance the doctor falls ill or departs, he shall leave another in his stead, so as not to hinder the birth at any time, thus encroaching upon the sick” [38].

Because of the medical advances made during the 18th–19th centuries, the healing process was less and less dependent upon divine intervention, making more important the role of the physician in the attempt to cure the patient (or at least alleviate his/her suffering). Thus, a Charter from 28 January 1778 issued by Constantin Moruzi said, “Nevertheless, the chosen physician shall be ready at all times, not only in tending for the prosperous, but also in curing the impoverished” [35]. The Doctor’s Decree of 1780 specified in turn, “arduously shall he go the great and small, in due time, to tend to each and every man of the common folk, as befitted, and until he is restored to steadiness” [36]. This accountability for physician’s acts generated numerous adjacent effects, such as regulating the pharmacies, which were forced to only sell drugs included in the Pharmacopeia of Vienna; the obligation to hold a certificate for practicing medicine, a certificate that could only be obtained after showing a proof of a license/PhD in medicine; the marked development of private practice, in which many nobles started employing their own physicians, and so on.

## 4. Discussion

Beginning with 1859, the year of the union between Moldavia and Wallachia, Turkish influence in the Romanian principalities decreased significantly, and medical profession became increasingly rooted in the West-European values. Thus, new codes of law were issued, a new health organization occurred, and the first medicine faculties and modern Physicians Colleges were founded, whose codes included elements of ethics, with all these aspects favoring the occurrence of a new modern medicine whose purpose may be properly summarized in the following paragraph written by Th Percival: “Physicians and Surgeons should minister to the sick with due impressions of the importance of their office; reflecting that the ease, the health, and the lives of those committed to their charge depend on their skill, attention, and fidelity. They should study, also, in their deportment, so to unite tenderness with steadiness, and condescension with authority, as to inspire the minds of their patients with gratitude, respect and confidence” [47].

## 5. Conclusions

The roots of the main concepts appertaining to medical ethics can be identified in the Moldova and Wallachia since the late Middle Ages. It has some important differences compared to Western Europe, mainly generated by its Eastern influences and a very high prevalence of Orthodoxy.

## Data Availability

Not applicable.
